# Photonic porous silicon as a pH sensor

**DOI:** 10.1186/1556-276X-9-420

**Published:** 2014-08-21

**Authors:** Stephanie Pace, Roshan B Vasani, Wei Zhao, Sébastien Perrier, Nicolas H Voelcker

**Affiliations:** 1 Mawson Institute, University of South Australia, GPO Box 247, Adelaide, South Australia 5001, Australia; 2 Key Centre for Polymer Colloids Room 351, School of Chemistry, University of Sydney, Sydney, NSW 2006, Australia; 3 Department of Chemistry, The University of Warwick, Coventry CV4 7AL, UK

**Keywords:** Porous silicon, pH-responsive polymer, RAFT polymerization, Color change sensor

## Abstract

Chronic wounds do not heal within 3 months, and during the lengthy healing process, the wound is invariably exposed to bacteria, which can colonize the wound bed and form biofilms. This alters the wound metabolism and brings about a change of pH. In this work, porous silicon photonic films were coated with the pH-responsive polymer poly(2-diethylaminoethyl acrylate). We demonstrated that the pH-responsive polymer deposited on the surface of the photonic film acts as a barrier to prevent water from penetrating inside the porous matrix at neutral pH. Moreover, the device demonstrated optical pH sensing capability visible by the unaided eye.

## Background

There is a need to develop rapid and biocompatible pH sensors to monitor changes in the wound-healing trajectory that are, for example, caused by bacterial infection or biofilm formation. Chronic wounds do not heal within 3 months, and are considered an important and costly medical issue in the world’s aging societies, imposing considerable pain, reduced mobility and decreased quality of life on the sufferers [1]. During the lengthy healing process, the wound is invariably exposed to bacteria that can colonize the wound bed and form biofilms. This alters the wound metabolism and brings about a change of pH [2]. Several recent studies have demonstrated an oscillation of the pH between 5.4 and 9, during a bacteria infection in the wounds [2,3].

Recently, significant research efforts have been devoted to pH sensors for the detection of pH variation in wound fluid [1]. These are typically based on dyes [4,5] or on inductive transducers [6] incorporated into wound dressings. For example, Trupp et al. have synthesized a series of hydroxyl-substituted azobenzene derivatives as indicator dyes for optically monitoring pH between 6 and 10 [4]. However, there are concerns over the biocompatibility of these dyes. Sridhar and Takahata have developed a micro-fabricated wireless pH monitor involving a pH-sensitive hydrogel intended to be imbedded into a wound dressing to track pH wirelessly. The authors observed changes in moisture level in a wound dressing in the pH range 2 to 7 [6]. The cost of this device may be a limiting factor for reduction to practice.

Simultaneously, materials with optical features such as the porous silicon (pSi) have been associated with pH-responsive polymers in order to detect variation of pH [7-9]. PSi is an attractive candidate to use as a sensor in contact with wound fluid because the material is highly biocompatible and well tolerated in vivo, even when implanted into the eye [10]. The material displays strong thin-film interference effects, which result in the appearance of Fabry-Pérot interference fringes [11]. In turn, multilayers of pSi of alternating high and low refractive indices result in a sharp photonic resonances [11]. Changes in the effective refractive index of pSi films cause a shift in the interference pattern or the position of the photonic resonance peak in multilayered pSi resonators, respectively [12-15]. Perelman et al. developed a pH sensor based on pSi modified with thermo- and pH-responsive hydrogel poly(*N*-isopropylacrylamide-co-acrylic acid). Shifts in the optical reflectivity spectra were observed when the pH changed from 7 to 4, due to changes in the dielectric composition and morphology of the hydrogel [9]. However, in this type of sensor, the change of refractive index caused by the polymer upon conformational switching is usually too small to induce a color change of the pSi film that is detectable without the aid of a spectrometer [16].

Here, we develop pSi-based photonic sensors to detect changes in pH. The originality of this sensor is to use a pH-responsive polymer plug that acts as a barrier to prevent the water from penetrating into the porous matrix at neutral pH. As the pH decreases, the polymer becomes hydrophilic, thus opening up the pores of the porous layer and enabling water penetration. The water penetration results in a conspicuous wavelength shift of the pSi reflector’s resonance, producing an optical signal visible to the unaided eye (Additional file 1).

## Methods

### Materials

2-Diethylaminoethyl acrylate (DEAEA) was obtained from Aldrich (Castle Hill NSW, Australia). The inhibitor was removed from DEAEA by passing the monomer two times over an inhibitor removal column from Sigma (Castle Hill NSW, Australia). 2,2′-Azobisisobutyronitrile (AIBN; Aldrich) was recrystallised from ethanol. 2-Propanoic acid butyl trithiocarbonate (PABTC) was supplied by Dulux (Rocklea, Australia). Toluene and tetrahydrofuran (THF; Aldrich) were of AR grade and were used as received.

### Synthesis of DEAEA polymer

PABTC (0.037 g, 0.155 mmol) was placed in a round bottom flask and AIBN (0.0051 g, 0.031 mmol) was added to it. To this mixture, DEAEA (4 g, 23.359 mmol) and toluene (1.33 g, 14.433 mmol) were added. The solution was homogenized by shaking at 0°C and deoxygenated by bubbling nitrogen through it for 20 min. The solution was placed in an oil bath at 65°C and polymerized for 24 h. After polymerization, the residual monomer and solvent was removed by precipitating the polymer in acetone. The polymer was dried under vacuum overnight.

Monomer conversion was calculated by ^1^H nuclear magnetic resonance (NMR), performed on a 200-MHz Bruker spectrometer (Bruker Daltonics, Victoria, Australia). Molecular weights and molecular weight distributions were determined by gel permeation chromatographic (GPC) analysis using tetrahydrofuran as an eluent (40°C, 1.0 mL/min). The instrument was previously calibrated with polystyrene standards (Polymer Laboratories, Church Stretton, UK) with molecular weights ranging from 580 to 7,500,000 g/mol.

### Photonic pSi film preparation

pSi films were prepared from single-crystal p-type silicon (boron doped, 0.0005 to 0.0011 ohm cm resistivity, <100 > orientation) at a modulated current density with a sine wave (between 11.36 and 28.4 mA/cm^2^, 21 s periodicity) for 477 s in a 1:1 (48%) aqueous hydrofluoric acid ethanol solution, to produce a rugate filter. After etching, the samples were thermally oxidized at 600°C for 1 h and then silanized with a solution of 4% of (3-aminopropyl)triethoxysilane (Sigma) in toluene for 1 h, to provide a hydrophilic and stable film in aqueous medium.

### Polymer spin coating

The polymer was deposed on the external surface of the pSi by spin coating, in a manner that the polymer acts as a barrier to prevent the ingress of water into the porous matrix. PDEAEA was dissolved in toluene (40 mg/mL) and was spin-cast on the pSi film at 3,000 rpm for 1 min. Three deposition cycles were carried out on the same sample in order to generate a thick layer of polymer. The sample was placed under vacuum for 12 h, in order to evaporate the solvent remaining in the surface.

### Fourier transform infrared spectroscopy

Fourier transform infrared (FTIR) spectroscopy was performed with a Hyperion (Bruker) coupled to the liquid nitrogen cooled Mercury-cadmium-telluride (MCT) detector, in attenuated total reflectance (ATR) mode. Background spectra were taken in air and all spectra were recorded with an aperture size of 3 mm, over the range of 650 to 3800/cm, at a resolution of 22/cm averaging 64 scans.

### Interferometry reflectance spectroscopy

Optical reflectivity spectra were obtained using an Ocean Optics USB2000 miniature fiber optic spectrometer (Ocean Optics, Inc, Dunedin, FL, USA). Samples were illuminated with a tungsten lamp.

### Contact angle measurements

Static water contact angles were measured both above and below the p*K*_a_ of pDEAEA. For measurements, a 3-μL drop of Milli-Q water (Millipore, Billerica, MA, USA), below the p*K*_a_ (pH 3 and pH 7) or above the p*K*_a_ (pH 9), was placed on the surface of a dry sample at room temperature and an image was captured using a Panasonic WV-BP550/G CCTV camera (Panasonic, Kadoma, Osaka, Japan). The contact angles were analyzed using ImageJ (version 1.41) software.

## Results and discussion

In order to design a pH-responsive polymer plug that acts as a barrier for water infiltrating into the pores of a pSi-based photonic film, poly(2-diethylaminoethyl acrylate) (pDEAEA) was chosen since the polymer’s pendant tertiary amine groups are deprotonated at pH > p*K*_a_ (p*K*_a_ of pDEAEA = 8.0) rendering the polymer hydrophobic [17]. When the pH decreases below the p*K*_a_, the amino groups present on polymer are quaternized and the polymer becomes hydrophilic [18]. Moreover, this polymer is not toxic and has been used in the past as a support for long-term human embryonic stem cell growth and pluripotency over a period of 2 to 6 months [19].

### Fabrication and characterization of pSi-pDEAEA films

PSi single films were prepared from single-crystal highly doped p-type silicon wafers using a sine wave-modulated current density between 11.4 and 28.4 mA/cm^2^ resulting in a rugate filter with a reflectivity peak of 540.0 nm and a full width at half maximum (FWHM) of 30 nm [20]. The porosity of the film was simulated from the reflectance spectra using the transfer matrix method [7,16,21], and oscillated between 68.5% and 78.3%. A thickness of 3,530 nm and pore sizes ranging from 25 to 45 nm in diameter were determined using scanning electron microscopy (data not shown).

**Figure 1:**
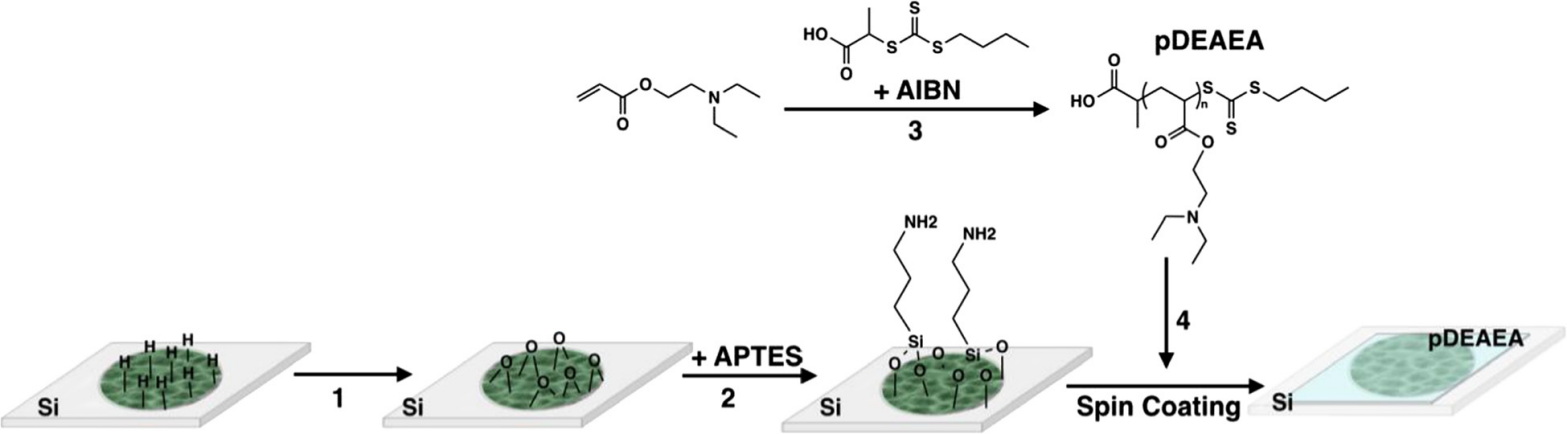
**Fabrication of pSi-pDEAEA composite films.** A piece of flat silicon is subjected to electrochemical etching using HF as an electrolyte followed by (1) thermal oxidation, (2) the oxidized pSi film is functionalized with the silane, (3) the DEAEA monomer is subjected to RAFT polymerization reaction, and (4) the pDEAEA is spin-coated onto the surface.

**Figure 2:**
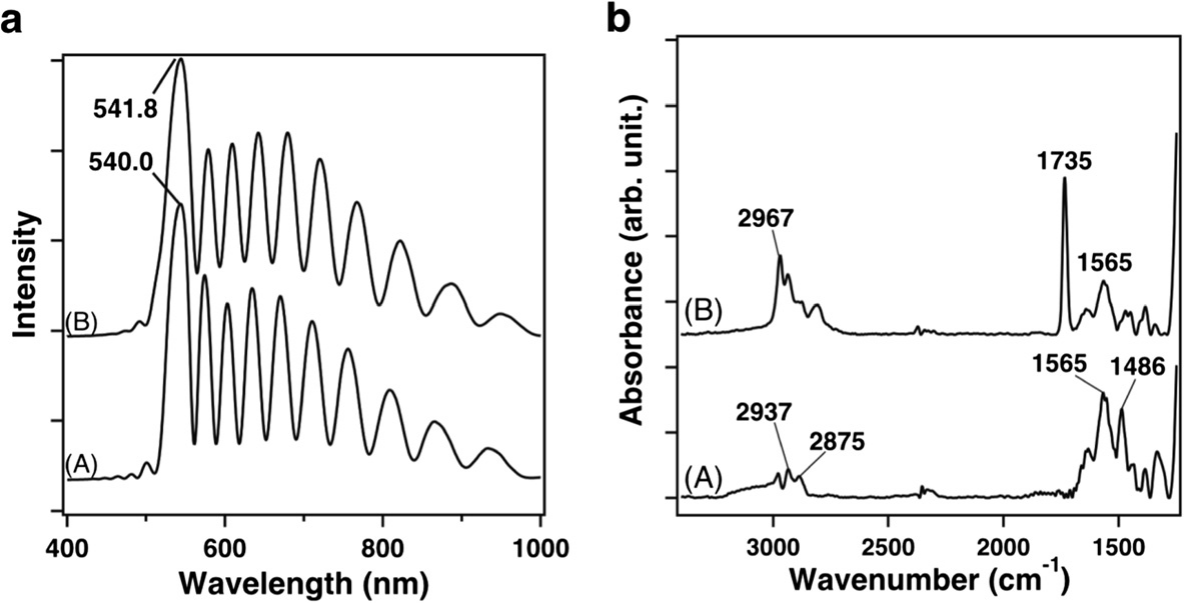
**Reflectance spectra of the oxidized pSi surface and FTIR-ATR spectra for pSi samples. (a)** Reflectance spectra of the oxidized pSi surface modified with silane (A) and of the pSi after spin coating of pDEAEA (B). **(b)** FTIR-ATR spectra for pSi samples modified with silane (A) and with a layer of pDEAEA spin-coated on the surface (B). The spectra were baseline-corrected.

After etching, the samples were thermally oxidized at 600°C for 1 h (Figure  1, step 1) and then silanized with a solution of 4% of (3-aminopropyl)triethoxysilane to afford a hydrophilic surface that is stable in aqueous media [16,22]. Reversible addition-fragmentation chain transfer (RAFT) polymerization was used to synthesize the pDEAEA following a published procedure (Figure  1, step 3) [18,23]. The resulting polymer had a molecular weight of 4,380 g/mol as determined by GPC. This polymer was deposited on the external surface of the pSi rugate filter by spin coating (Figure  1, step 4), in a manner that the polymer acts as a barrier to prevent the ingress of water into the porous matrix.In order to test the reliability of using the optical properties of pSi rugate filters and the penetration of the polymer inside the pores, the white-light reflectance spectrum from the pDEAEA-covered pSi film modified with silane was recorded and compared with the silane-functionalized pSi film without polymer. The spectrum obtained from the silanized pSi displays a sharp resonance at a wavelength of 540.0 nm (Figure  2a, trace A). Figure  2a (trace B) shows the reflectance spectrum at the same spot after spin coating of the polymer. The rugate peak is observed at a wavelength of 541.8 nm, very similar to the resonance observed for the control, therefore confirming that the polymer has not penetrated into the pores to a significant extent. The intensity of the reflectance spectrum of the sample modified with pDEAEA is slightly smaller (~1.3 times smaller) than the one observed for the control. Had the pores been filled with polymer during spin coating, the resonance peak would be expected to red shift by approximately 111 nm according to a simulation using the transfer matrix method. We conclude from these observations that the presence of the pDEAEA does not obstruct the optical spectrum of the pSi reflector. In addition, the lack of a significant change in the wavelength of the rugate peak before and after the polymer layer deposition confirms that pDEAEA does not infiltrate the porous layer.

The decrease in intensity of the pSi-pDEAEA reflectance spectrum is also consistent with the presence of polymer layer on the top of the pSi film since the amplitude of the reflectance spectrum is correlated to the index contrast at the interfaces [16,24]. Here, three interfaces are present: air-pDEAEA, pDEAEA-pSi, and pSi-Si bulk. In the literature, the relationship between the thickness and the refractive index of the layers deposited at the surface of the pSi and the variation in amplitude in the reflectance spectra is well established [16,25]. Here, the transfer matrix method from the program SCOUT was used to calculate a layer thickness of pDEAEA on the top of the pSi film. Indeed, for the calculus, the reflectance spectrum of the control was used as a reference and the thickness of the polymer layer was the parameter that was adjusted in order to obtain a best fit between the reflectance spectrum of the control (trace A) and the reflectance spectrum of the pSi-pDEAEA (trace B). For the calculus, we assumed that the refractive index of the pDEAEA was similar to the poly(*N*-*N* diethylaminoethyl methacrylate) (*n* = 1.51) [26]. A layer thickness of 70 nm of pDEAEA deposited on the surface of the pSi was obtained.

FTIR spectroscopy was used to confirm the result obtained with the interferometry reflectance analysis and to characterize the chemical groups present at the surface of the pSi rugate filters (Figure  2b), after thermal oxidation and silanization (A) and after spin coating of the pDEAEA (B). For the two spectra, the measurements were performed in the attenuated total reflection (ATR) mode. Spectrum A of Figure  2b exhibits bands at 1,486, 2,875, and 2,937/cm, assigned to the deformation and stretching (symmetric and asymmetric) vibrational modes of the aliphatic C-H_2_ groups, respectively. The presence of band at 1,565/cm was attributed to the deformation vibrational mode of the N-H bond. The presence of the specific bands of the C-H_2_ groups and the N-H bond are evidence of successful silanization. In spectrum B, the presence of an intense band at 1,735/cm was assigned to the ν(C = O) stretching vibrational mode of the ester bonds of the polymer. Additionally, the band at 2,967/cm was assigned to the stretching vibrational mode of the C-H_3_ groups and the bands assigned to tertiary amino moieties (2,700 to 2,850/cm) were present in the spectrum, confirming the presence of a polymer layer on the surface [27].

### pH-responsiveness on the pSi-pDEAEA film

**Figure 3:**
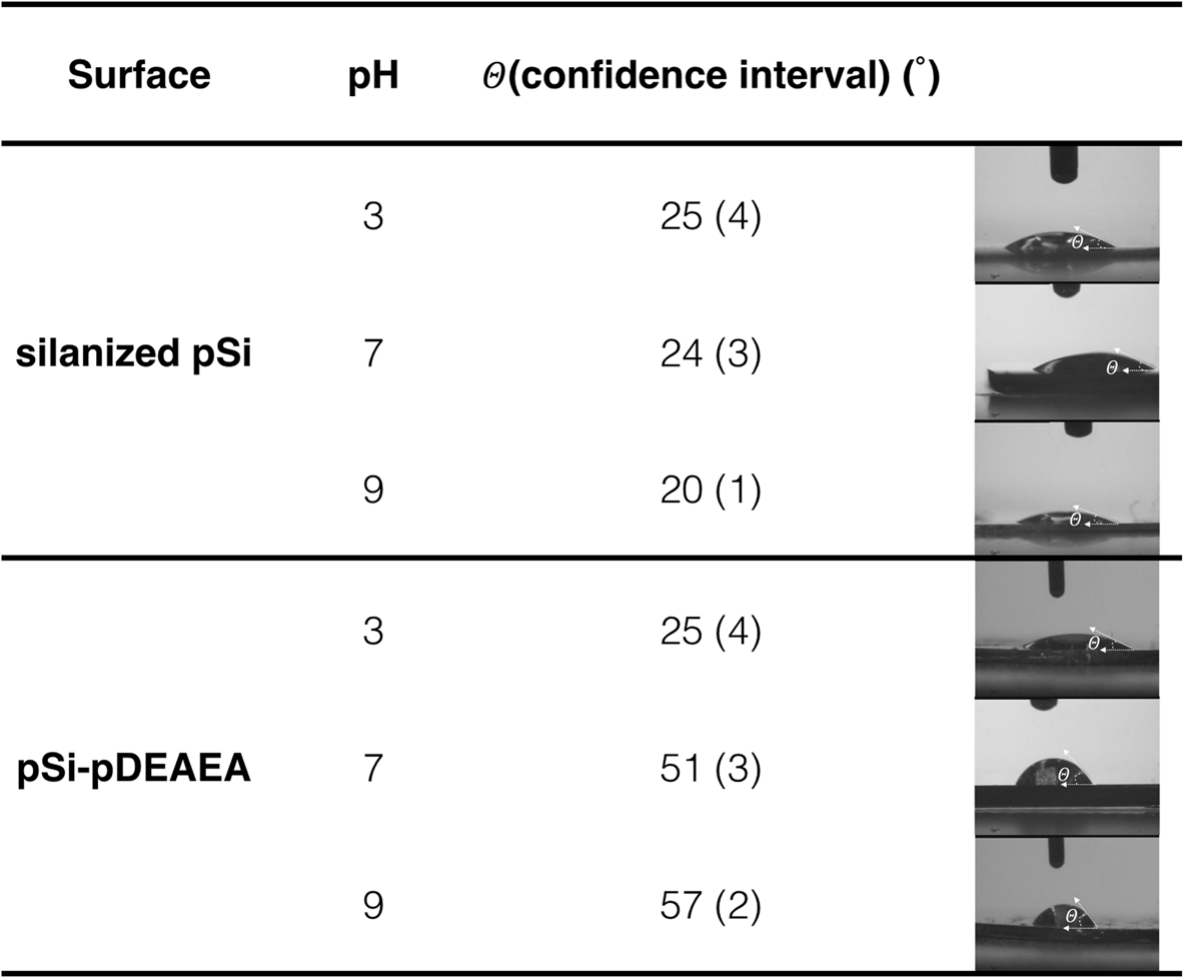
**Water contact angles at different pH values below and above the p** ***K***_**a **_**of the polymer.**

The wettability of the silanized pSi and the pSi-pDEAEA films were compared at three different pH (3, 7, and 9) below and above the polymer’s p*K*_a_ using water contact angle measurements (Figure  3). Usually, contact angle measurements are considered for ideal flat surfaces that are traditionally defined as being smooth, rigid, chemically homogeneous, and non-reactive [28]. In the case of solid surfaces presenting roughness or chemical heterogeneity, quantitative interpretation of contact angle values is not straightforward [29]. However, we are only interested in qualitative differences. The contact angles at pH 3 and pH 9 for the pSi-pDEAEA sample differed by almost 30° (from a mean of 25.0° to 57.2°). For silane-functionalised pSi, the contact angles were 25.2° at pH 3 and 20.3° at pH 9. At pH 3, there was no significant difference observed between the silanized sample and the pSi-pDEAEA, but a significative change was noticed at pH 9. The difference in contact angle between the control and the pSi-pDEAEA films at pH 9 can be explained by the pH-dependent wettability properties of the polymer. At a pH above the p*K*_a_, the polymer is hydrophobic since the amine groups are deprotonated and the polymer undergoes intramolecular hydrogen bonding. Similar results are observed for both surfaces when they are exposed to a drop of water at pH 7. The contact angle measured for the pSi-pDEAEA sample at pH 7 is 51.9°. The hydrophobicity of this surface at pH 7 can be explained by a decrease of the positive charges on the amino groups presented on polymer. When the pH is close to the p*K*_a_ value of the polymer, a larger fraction of amino groups are deprotonated, explaining that the surface is more hydrophobic at pH 7 than at pH 3, since the condition are very close to the p*K*_a_ value [30]. Our experiment confirms that the polymer maintains these switchable properties when spin-coated onto pSi.

The efficiency of the polymer to act as a barrier and the change of color of the pH sensor were tested by placing a drop of water of different pH (pH 3 and pH 7) on dry rugate filters of pSi-pDEAEA and silanized pSi as a control. The experiments were performed at pH 7, in order to mimic the physiological condition.

**Figure 4:**
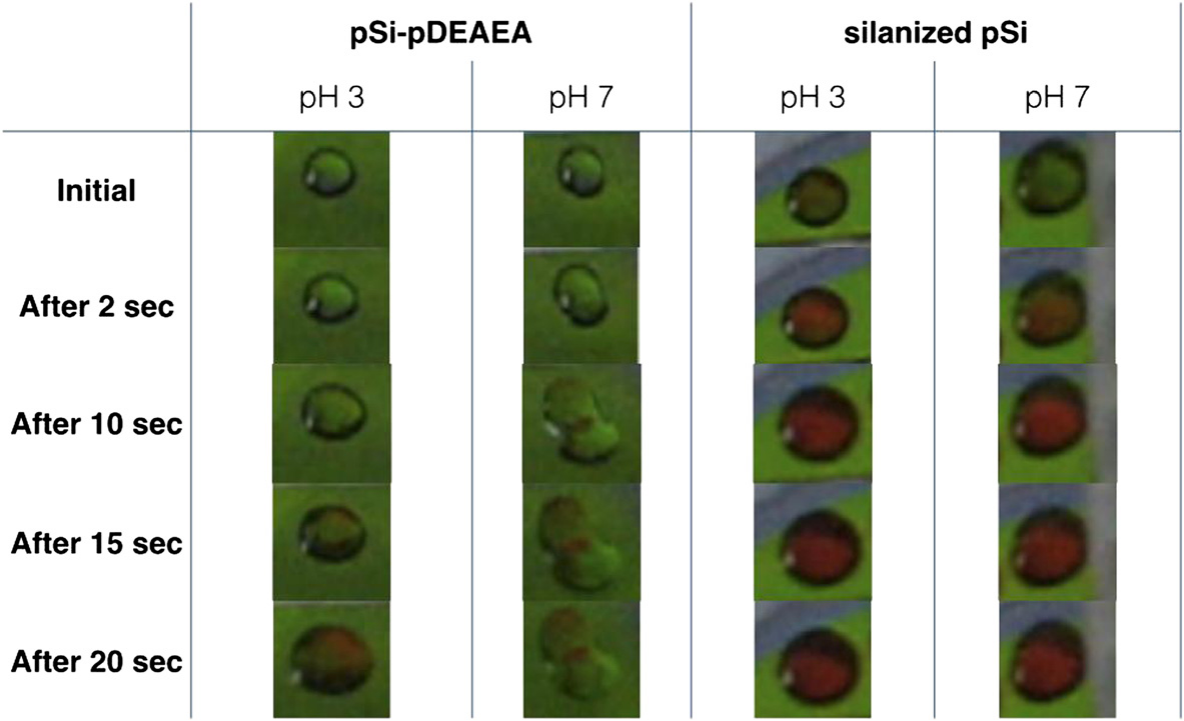
Photographs of silanized pSi and pSi-pDEAEA rugate films that display changes in optical color when exposed to water.

In air, both dry films appeared green due to the position of the photonic resonance. Figure  4 shows the image of the samples with water droplets over time. The control sample turned red in a matter of seconds after being exposed to the water. In contrast, the pSi-pDEAEA remains green underneath the water droplet at pH 7. The change of color observed for the control, can be explained by a variation of refractive index inside the porous matrix. At the beginning of the experiment, the pores are filled with air (*n*_air_ = 1) and the samples appear green. After the deposition of water droplet on the surface, the water (*n*_water_ = 1.33) penetrates inside the pores and the position of the photonic resonance shifts towards the red. The green color observed for the pSi-pDEAEA even after being exposed to the water confirms the presence of the polymer on the external part of the surface acting as a barrier to water infiltration.

After longer incubation time, the color shifts from green to red for the pSi-pDEAEA upon exposure to a water droplet at pH 3. In contrast, the pSi-pDEAEA sample with the water droplet of pH 7 is still green. These results show that the polymer resists water infiltration at pH 7. The protonated polymer at pH 3 allows water to soak into the porous layer, giving rise to a shift in the photonic resonance.

## Conclusions

We have developed an optical pH sensor based on a photonic pSi film where a pH-responsive polymeric layer on top of the porous layer modulates ingress of water into the layer. The pH-responsive polymer pDEAEA was chosen, synthesized by RAFT polymerization, and spin-coated on pSi rugate filters. FTIR spectroscopy, interferometry reflectance spectroscopy, and water contact angle measurements were used to confirm the exclusive presence of the polymer at the external surface of the rugate filter. After exposing the pSi-pDEAEA to water droplets of different pH, the role of the polymer as a barrier was demonstrated in contrast to a control sample lacking the polymer. Penetration of water into the porous layer, associated to a change of color of the sample, only occurred at low pH. Our study therefore provides proof-of-principle that photonic pSi can be used to detect pH changes in aqueous medium. This sensor can potentially be incorporated into wound dressings and used to report on acidification of chronic wound fluid as a result of bacterial infection through a color change that is visible to the unaided eye. Such a device would provide fast wound diagnostics to practitioners and nurses.

## Competing interests

The authors declare that they have no competing interests.

## Authors’ contributions

SPa and WZ carried out the polymer synthesis and the polymer characterization. SPa carried out the porous silicon synthesis and the characterization and drafted the manuscript. RV participated in the samples characterization. SPa, SPe, and NV conceived of the study, and participated in its design and coordination. NV helped to draft the manuscript. All authors read and approved the final manuscript.

## Authors’ information

SPa is research associate at the Mawson Institute from the University of South Australia. RV is a PhD student at the Mawson Institute from the University of South Australia. WZ is a PhD student at the Key Centre for Polymer Colloids in the School of Chemistry from University of Sydney. SPe is a full professor in the Department of Chemistry from the University of Warwick in UK. NV is a full professor from the Mawson Institute from the University of South Australia.

## Supplementary Material

Additional file 1 **Porous silicon photonic films.** Porous silicon photonic films modified with the pH-responsive polymer poly(2-diethylaminoethyl acrylate) are employed to detect a change in pH, through a color change visible by the unaided eye. Click here for file
