# Supplementary material for: Photonic porous silicon as a pH sensor
Source: Nanoscale Res Lett. 2014 Aug 21;9(1):420. doi: 10.1186/1556-276X-9-420 (PMC4147967; doi:10.1186/1556-276X-9-420)

Porous silicon photonic films modified with the pH-responsive polymer : poly(2-diethylaminoethyl acrylate) are employed to detect a change in pH, through a color change visible by the unaided eye.


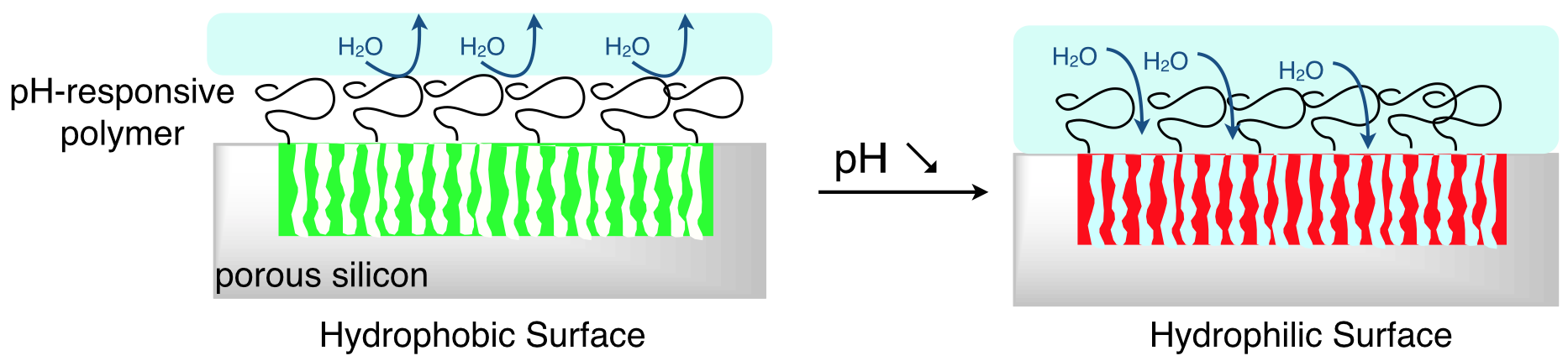

Supplement: Additional file 1 — Porous silicon photonic films. Porous silicon photonic films modified with the pH-responsive polymer poly(2-diethylaminoethyl acrylate) are employed to detect a change in pH, through a color change visible by the unaided eye. [file 1556-276X-9-420-S1.docx]
